# Supplementary material for: The Transcriptomic Analysis of the Response of Pinus massoniana to Drought Stress and a Functional Study on the ERF1 Transcription Factor
Source: Int J Mol Sci. 2023 Jul 5;24(13):11103. doi: 10.3390/ijms241311103 (PMC10342239; doi:10.3390/ijms241311103)
Supplement: Supplementary file 1 [file ijms-24-11103-s001.zip › Supplemental images.pdf]

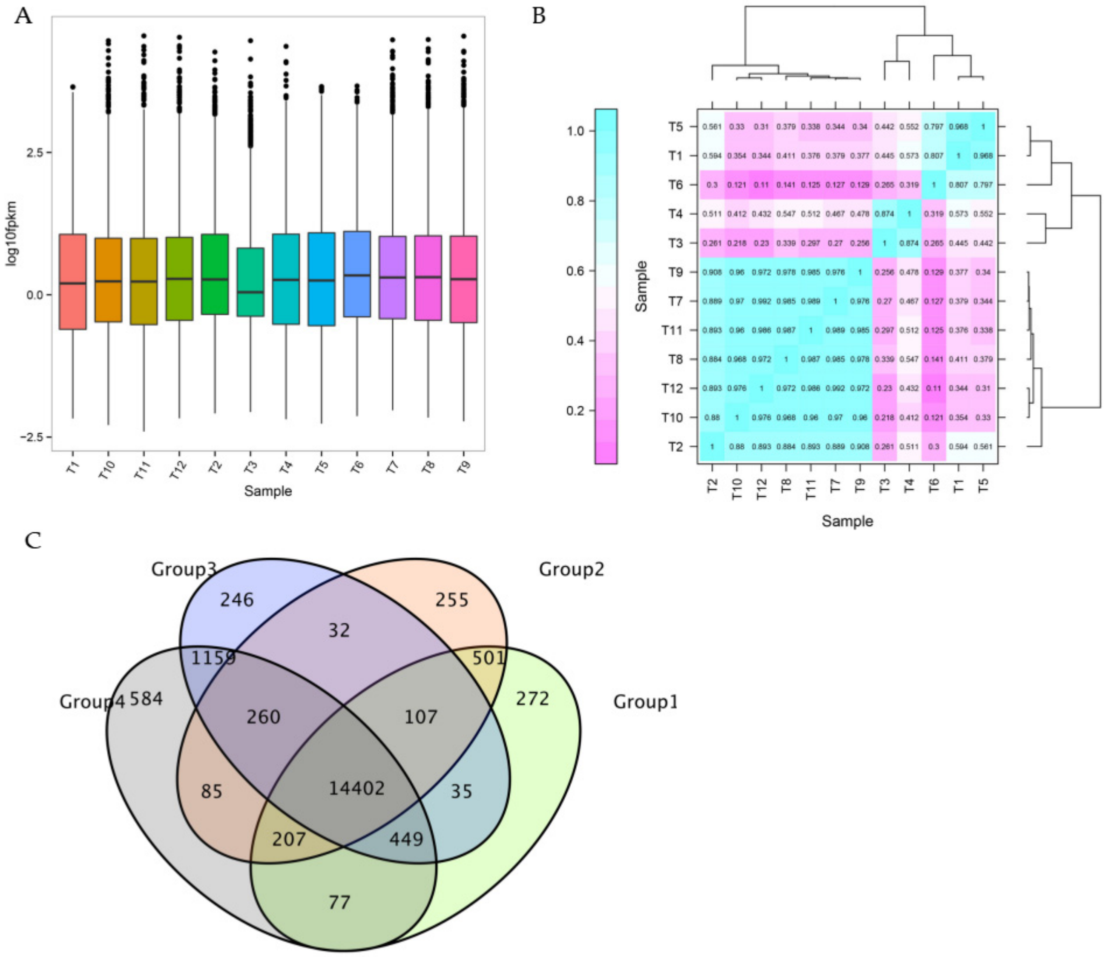

Supplemental Figure S1. The analysis of the expressed genes. (A) Boxplot of the gene expression distribution for each sample. (B) Principal component analysis. (C) Venn diagram of four samples.

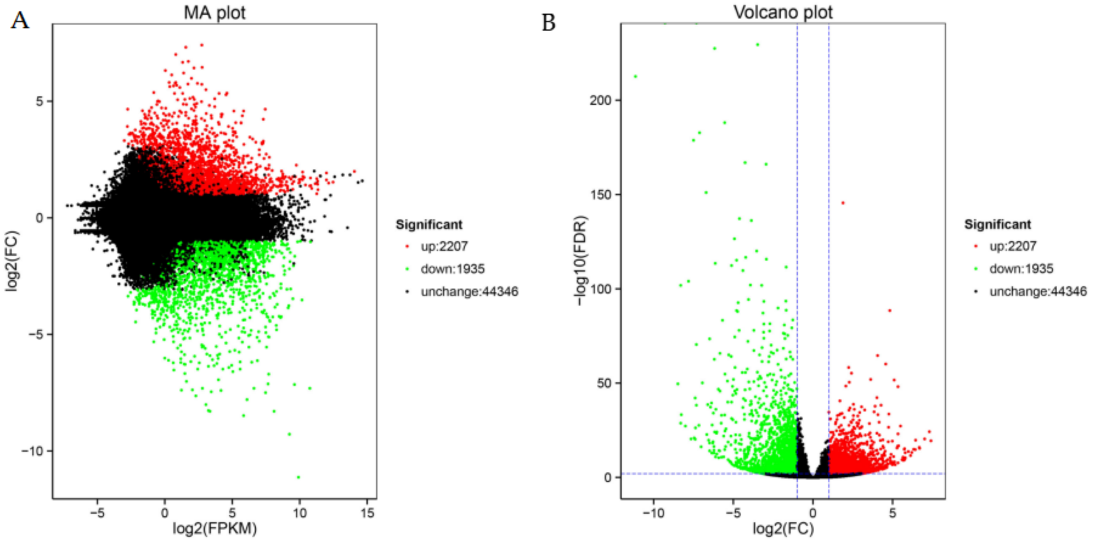

Supplemental Figure S2. MA(A) and volcano plots (B) of RNA-seq data of the G1 vs G4 pairwise comparison.

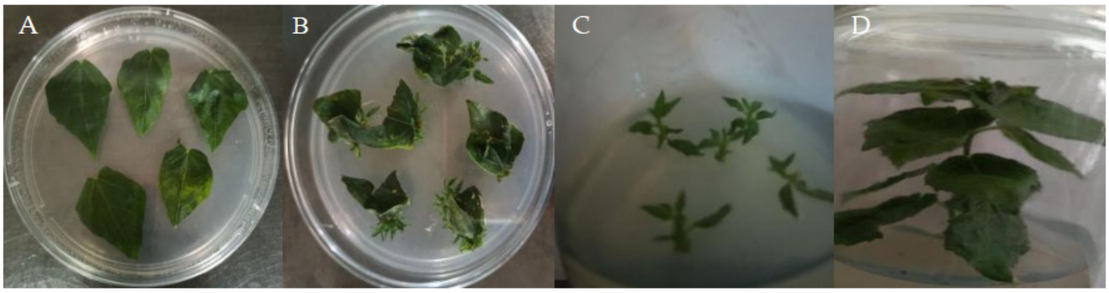

Supplemental Figure S3. Acquisition of the transgenic poplar poplar over-expressing *PmERF1*. (A) Co-culture medium. (B) Bud culture medium. (C) Bud elongation medium. (D) Rooting medium.
